# High Dynamic Weak Signal Tracking Algorithm of a Beidou Vector Receiver Based on an Adaptive Square Root Cubature Kalman Filter

**DOI:** 10.3390/s21206707

**Published:** 2021-10-09

**Authors:** Na Li, Shufang Zhang, Yi Jiang

**Affiliations:** 1 Information Science and Technology College, Dalian Maritime University, Dalian 116026, China; genuine_666@dlmu.edu.cn (N.L.); j_y@dlmu.edu.cn (Y.J.); 2 Physics and Electronic Information College, Hulunbuir University, Hulunbuir 021008, China

**Keywords:** Beidou, vector tracking, high dynamics, weak signal, ASRCKF, SRCKF, innovation

## Abstract

Compared with a scalar tracking receiver, the Beidou vector tracking receiver has the advantages of smaller tracking errors, fast loss-of-lock reacquisition, and high stability. However, in extremely challenging conditions, such as highly dynamic and weak signals, the loop will exhibit a high degree of nonlinearity, and observations with gross errors and large deviations will reduce the positioning accuracy and stability. In view of this situation, based on the concepts of cubature Kalman filtering and square root filtering, a square root cubature Kalman filtering (SRCKF) algorithm is given. Then, combining this algorithm with the concept of covariance matching based on an innovation sequence, an adaptive square root cubature Kalman filter (ASRCKF) algorithm is proposed. The algorithm was verified, and the tracking performance of the vector locking loop (VLL) realized by the algorithm was compared with the SRCKF VLL and the ASRCKF scalar locking loop (SLL). The simulation results show that, regardless of whether in a highly dynamic weak signal environment or in a general situation where the signal-to-noise ratio is higher than the tracking threshold, the tracking accuracy and stability of the ASRCKF VLL are higher than those of the SRCKF VLL and the ASRCKF SLL, the three-dimensional position error of the ASRCKF VLL does not exceed 36 m, and the three-dimensional velocity error does not exceed 3.5 m/s.

## 1. Introduction

With the continuous improvement of the Beidou satellite navigation system, navigation and positioning applications based on the Beidou system are also gradually being developed. However, in various highly dynamic applications, such as cruise missiles and high-orbit space, the carrier frequency of the signal received by the receiver will contain a large Doppler shift and rate of change so that the traditional scalar receiver cannot complete the tracking and positioning. At this time, if the correlation between the signal channels can be used to assist the weak signal channel with the tracking result of the strong signal channel, then the loop can continue to track the signal without losing lock. This concept of using the correlation between signal channels to further improve the tracking performance of the receiver is called vector tracking.

The concept of vector tracking was proposed as early as 1996 by Spilker et al. [1]. After entering the 21st century, vector tracking technology has been given importance by scholars and developed rapidly. Pany et al., from the University of Munich, Germany, were the first to give a detailed structure of the vector delay locking loop (VDLL), vector frequency locking loop (VFLL), and their software receiver form [2,3]. Reference [4] introduced a variant of the vector delay locking loop tracking algorithm for tracking L1 civil GPS signals. The VDLL architecture uses a single extended Kalman filter to predict the phase of the satellite PRN code and track the user’s position, speed, and clock state. In addition, each channel uses a series of independent Kalman filters to track the satellite carrier signal. Then, the advantages of the architecture based on the vector/Kalman filter over traditional methods can be explained. References [5,6] discussed the continuous tracking of GPS signals by vector tracking loops in the case of weak signals and compared them with the traditional scalar tracking method.

Domestic scholars and doctors have also conducted in-depth research on vector tracking rings. Zhao Sihao from Tsinghua University and Dennis Akos from the University of Colorado, USA, together presented the open-source GPS/GNSS vector tracking loop code. Experiments have proven that the proposed VLL structure is superior to SLL in terms of the accuracy of the navigation solution and the ability to cover GPS signal interruptions [7]. In addition, Zhu Zhenzhen from the National University of Defense Technology gave a nonlinear observation equation of the VDLL, linearized the established model, and realized the VDLL with an extended Kalman filter (EKF). The research results show that the vector code loop can maintain lock even when several channels lose lock momentarily. This advantage makes the vector code loop suitable for highly dynamic and weak signal environments [8].

The abovementioned research on the vector tracking loop provides a new idea for the tracking of highly dynamic weak signals in a GPS system. For the nonlinear model of the vector tracking loop, the more commonly used method is the EKF algorithm. In highly dynamic applications, the high degree of nonlinearity caused by high dynamic stress will incur greater errors if the EKF algorithm is used. In this case, nonlinear filtering algorithms such as unscented Kalman filters (UKF), particle filters (PF) [9], strong tracking Kalman filters [10], and cubature Kalman filters (CKF) are needed. Among them, the cubature Kalman filter algorithm is a newly proposed algorithm [11] that is derived from rigorous theory and can be accurate to the third order of Taylor expansion. Unlike the EKF algorithm, there is no error caused by the linearization process. Compared with the unscented Kalman filter, it reduces one sampling point. However, CKF has the characteristics of numerical instability and a large amount of computation in the recursive process, and once the initialization parameters are set, the parameters cannot be adjusted in real time as the environment changes [12,13]. In response to the above problems, this paper draws on the ideas of innovation [14] and square root filtering [15] on the basis of cubature Kalman filtering to estimate the covariance of measurement noise in real time. At the same time, in the filtering process, the recursive update is directly performed in the form of the square root of the covariance matrix to reduce the computational complexity and to ensure the nonnegative definiteness of the covariance matrix, which effectively avoids the divergence of the filter.

This article mainly solves the tracking problem of high dynamic weak signals. In order to adapt to the Doppler frequency shift of the carrier signal in a high dynamic environment, the traditional receiver needs to increase the loop bandwidth, and the increase in the bandwidth will introduce noise interference. On the contrary, if the bandwidth is not increased, the carrier Doppler frequency shift is likely to exceed the tracking range of the loop. Therefore, the loop bandwidth can only be chosen as a compromise. In order to improve the accuracy of signal tracking, this article no longer uses the traditional scalar tracking loop but uses a vector tracking loop to make full use of the information correlation between each channel. The navigation filter in the loop tracks the carrier and the C/A code while performing navigation calculations on parameters such as position, speed, and clock error. Secondly, under high dynamic conditions, the carrier signal frequency, the first and second derivatives of the frequency, and other parameters not only change rapidly but also have strong nonlinearity [16]. In this case, the navigation filter needs to have a strong nonlinear filtering performance. For this, this paper uses the cubature Kalman filter algorithm based on the spherical radial criterion and combines the algorithm with the square root filter and the innovation covariance idea to propose an adaptive square root cubature Kalman filter algorithm, which realizes real-time adjustment of the measurement noise covariance matrix according to changes in the environment so that the tracking loop can always keep the signal locked even in the challenging environment of high dynamic and weak signal, and the RMS frequency estimation error, position error, and velocity error can be maintained at a better level.

This paper is organized as follows: Section 2 gives the operating mechanism of the vector tracking loop, the process equation, and the measurement equation of the loop in high dynamics. Through the analysis of the specific form of the measurement equation, the nonlinear relationship between the measurements and the states is given. In this nonlinear relationship, an adaptive square root cubature Kalman filter algorithm is proposed for estimating position, velocity, acceleration, and other parameters as accurately as possible, as well as for real-time processing of measurement noise items. Section 3 gives the specific design and implementation process of the ASRCKF algorithm. Section 4 uses the navigation signal simulator to generate the navigation signals needed for the experiment and verifies the proposed algorithm in the Beidou vector software receiver. Section 5 gives a summary of the whole paper.

## 2. Vector Tracking Loop Operation Mechanism

In a traditional receiver, each tracking channel is independent of the other; each channel completes the tracking of the satellite signal; measures the pseudorange and pseudorange rate of the corresponding satellite; and then obtains the user’s position, speed, and receiver clock difference through the navigation solution module. However, because each tracking channel of the receiver completes the tracking and positioning of the same user, there is correlation between the channels, while the traditional receiver, that is, the receiver using the scalar tracking method, ignores the correlation between the channels. In contrast, the vector tracking loop simultaneously processes the signals from all channels to give the navigation solution, and the common processing of all channels can make use of more information hidden in the signals so that the channels can help each other, thus improving the performance in a low carrier-to-noise ratio environment.

**Figure 1:**
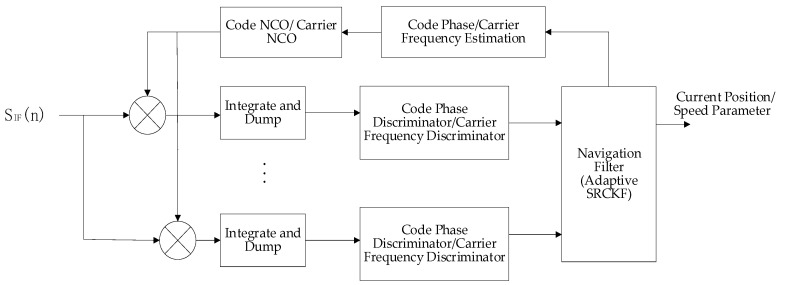
Vector tracking loop structure block diagram.

Vector tracking technology is a very promising emerging receiver technology. Its operating mechanism is shown in Figure 1.

First, the IF signal is multiplied and correlated with the local carrier and local C/A code, and the high frequency and noise in the signal are filtered through the integral cleaner to improve the carrier-to-noise ratio. The code phase error and carrier frequency error containing noise are extracted by the code phase discriminator and carrier frequency discriminator and input into the navigation filter to obtain the error between the estimated value and the true value of the parameters, such as position and speed. The adaptive SRCKF algorithm is used to obtain the modified values of the estimated values of each parameter at the current time, output the estimated values of each parameter at the next time, obtain the code phase and carrier frequency estimation through geometric projection, and adjust the local code/carrier generator to output the local carrier and pseudocode to enter the next cycle.

When the receiver is in a highly dynamic state, its vector tracking loop model will be very different from that of the nonhigh dynamic state. It is assumed that the velocity, acceleration, and jerk vector of the receiver in a highly dynamic state are as follows: $V=\left[V_{x}\;V_{y}\;V_{z}\right]$, $V^{\prime }=[{V_{x}}^{\prime }\;{V_{y}}^{\prime }\;{V_{z}}^{\prime }]$, and $V^{\prime \prime }=\left[{V_{x}}^{\prime \prime }\;{V_{y}}^{\prime \prime }\;{V_{z}}^{\prime \prime }\right]$; then, the process equation is as follows [17]

(1) $${\left[X_{k}\right]}^{2}={\left[\begin{array}{ccc} I_{3} & TI_{3} & \frac{1}{2}T^{2}I_{3}\\ 0_{3} & I_{3} & TI_{3}\\ 0_{3} & 0_{3} & I_{3} \end{array}\right]}^{2}{\left[X_{k-1}\right]}^{2}+{\left[\begin{array}{c} \frac{1}{2}T^{2}I_{3}\\ TI_{3}\\ I_{3} \end{array}\right]}^{2}{\left[W_{k-1}\right]}^{2}$$

where $X_{k}={\left[L_{kx}\;L_{ky}\;L_{kz}\;V_{kx}\;V_{ky}\;V_{kz}\;V_{kx}^{\prime }\;\;V_{ky}^{\prime }\;\;V_{kz}^{\prime }\;\right]}^{T}$, $W_{k-1}=\left[V_{kx}^{\prime \prime }\;V_{ky}^{\prime \prime }\;V_{kz}^{\prime \prime }\right]$ is the state disturbance noise.

If the output of the code phase discriminator and carrier frequency discriminator is selected as the observation, the system measurement equation is as follows [7]:

(2) $${\left[Z_{k}\right]}^{2}={\left[h\left(\Delta X_{k}\right)\right]}^{2}+{\left[U_{k}\right]}^{2}$$

where

(3) $$Z={\left[z_{code,1,k}\;\;z_{carrier,1,k}\;\;z_{code,2,k}\;\;z_{carrier,2,k}\;\;\cdots \;\;z_{code,n,k\;\;}z_{carrier,n,k}\right]}^{T}$$

where

(4) $$z_{code,j,k}=e_{x,j,k}\Delta x_{k\;}+e_{y,j,k}\Delta y_{k}+e_{z,j,k}\Delta z_{k}+\Delta t_{b,k}+v_{code,j,k}$$

(5) $$z_{carrier,j,k}=e_{x,j,k}\Delta v_{x,k\;}+e_{y,j,k}\Delta v_{y,k}+e_{z,j,k}\Delta v_{z,k}+\Delta t_{d,k}+v_{carrier,j,k}$$

In the above expression, subscript ‘*j*’ represents the *j*th satellite, ‘*k*’ represents the time epoch of the data, and ‘$a_{j}$’ is the line-of-sight direction unit vector between the receiver and the *j*th satellite. It is assumed that the position coordinates of the user at time *k* are ($x_{k}$, $y_{k},z_{k}$). The position of the *j*th satellite at time *k* is ($x_{j,k}$, $y_{j,k},z_{j,k}$) [7], then

(6) $$e_{x,j,k}=\frac{x_{j,k}-x_{k}}{q_{j,k}}$$

(7) $$e_{y,j,k}=\frac{y_{j,k}-y_{k}}{q_{j,k}}$$

(8) $$e_{z,j,k}=\frac{z_{j,k}-z_{k}}{q_{j,k}}$$

where, $q_{j,k}$ is

(9) $$q_{j,k}=\sqrt{{\left(x_{j,k}-x_{k}\right)}^{2}+{\left(y_{j,k}-y_{k}\right)}^{2}+{\left(z_{j,k}-z_{k}\right)}^{2}}=\;\sqrt{{\left(x_{j,k}-x_{k-1}-\Delta x_{k}\right)}^{2}+{\left(y_{j,k}-y_{k-1}-\Delta y_{k}\right)}^{2}+{\left(z_{j,k}-z_{k-1}-\Delta z_{k}\right)}^{2}}$$

From expressions (2), (9), it can be seen that there is a nonlinear relationship between measurements $Z_{k}$ and states $\Delta X_{k}$.

In the highly dynamic state of the receiver, the vector tracking loop has better tracking performance than scalar tracking. For example, it can be assisted by strong signals when tracking weak signals, and fast reacquisition can be achieved when the signals are blocked. However, it can be seen from the established highly dynamic vector tracking model that there is a highly nonlinear relationship between the measurements and the states. At this time, it is necessary to use not only a high-performance nonlinear filtering algorithm to estimate the states as accurately as possible but also real-time processing of the measurement noise to adapt to the high-frequency real-time changes of various parameters in a highly dynamic environment. Therefore, this paper adopts a new nonlinear filtering algorithm—cubature Kalman filtering (CKF)—proposed in recent years, which uses a set of sampling points to approximate the state value and covariance of the nonlinear system based on the third-order spherical radial criterion, has a strict and complete theoretical basis of numerical integration, and is superior to nonlinear filtering algorithms such as UKF and PF in terms of computational complexity and reliability. At the same time, to avoid filtering divergence caused by rounding errors in practical applications, the square root filtering idea is applied to CKF and further combined with adaptive filtering; an adaptive square root cubature Kalman filtering algorithm is proposed to realize real-time signal tracking.

## 3. Realization of Adaptive Square Root Cubature Kalman Filter Algorithm in Vector Receiver

### 3.1. Cubature Kalman Filter

First, the volumetric Kalman filter is a filtering method based on the spherical-radial criterion. Suppose there is the following vector function integral in the Cartesian coordinate system [18]:

(10) $$I\left(g\right)=\int_{R^{n}}^{\;}g\left(x\right)e^{-x^{T}x}dx$$

among them, if $x=ry,\;y^{T}y=1$, the integral in the Cartesian coordinate system is converted into the following spherical-radial coordinate integral:

(11) $$I\left(g\right)=\int_{0}^{\infty }r^{n-1}e^{-r^{2}}dr\int_{U_{n}}^{\;}g\left(ry\right)d\delta \left(y\right)$$

Assuming that the spherical integral $\int_{U_{n}}^{\;}g\left(ry\right)\;d\delta \left(y\right)$ is numerically calculated by the $L_{s}$ point spherical rule, and the radial integral $\int_{0}^{\infty }S\left(r\right)r^{n-1}e^{-r^{2}}dr$ is numerically calculated by the $L_{r}$ point Gaussian integral rule, Formula (12) can be approximated by the spherical-radial rule as [18]

(12) $$I\left(g\right)\approx \int_{0}^{\infty }r^{n-1}e^{-r^{2}}\overset{L_{s}}{\underset{i=1}{\sum }}w_{s,i}g\left(ry_{i}\right)dr\;\approx \overset{L_{r}}{\underset{j=1}{\sum }}\overset{L_{s}}{\underset{i=1}{\sum }}w_{r,j}w_{s,i}g\left(r_{j}y_{i}\right)\;=\sum_{i=1}^{L}w_{i}g\left(\xi_{i}\right)$$

When CKF processes nonlinear equations, it generates a point set based on the mean value and covariance of the prior probability density distribution of the system state and performs nonlinear propagation of the sampling points to approximate the Gaussian integral in nonlinear Gaussian filtering [19]. The cubature Kalman filter is similar to the Kalman filter, and it is also divided into two processes: prediction and correction. The implementation steps are as follows [11]:

(1) Prediction
  - Initializing state quantity, error covariance, process noise, and measurement noise;
  - Calculate and propagate volume points;
  - Calculate the predicted value of states and error covariance.
(2) Amendment
  - Calculate and propagate volume point;
  - Calculate the predicted value of the measurement;
  - Calculate measurement error covariance and cross-covariance;
  - Gain update, states, and error covariance update.

This process does not include linearization errors, and there is no need to calculate the Jacobian matrix; however, to ensure the stability of the filtering process value, the state error covariance matrix needs to have two properties of symmetry and positive definiteness. The iterative process of the CKF algorithm may destroy the positive definiteness and symmetry of the covariance and cause numerical instability. For this reason, this article is based on the square root filtering idea of the Kalman filter to propose the square root cubature Kalman filter algorithm, the algorithm directly uses the process noise, measures the square root factor of the noise and error covariance matrix, and generates a set of volume points to spread throughout the nonlinear system model. In each iteration, the square root value of the error covariance will be updated and propagated through the even number of cubature points generated.

### 3.2. Square Root Cubature Kalman Filter

SRCKF contains two parts: time update and measurement update. The main difference from the CKF algorithm is that the QR decomposition is introduced during filtering, and the recursive update is directly performed in the form of the square root of the covariance matrix. The specific steps are as follows [11]:

1. Time updating

Assuming the state estimation and state estimation error covariance at time epoch *k* − 1 are ${\hat{X}}_{k-1|k-1}$ and $P_{\;k-1|k-1}$, then the posterior probability distribution is $P\left(X_{k-1}|Z_{k-1}\right)=N\left(X_{k-1};{\hat{X}}_{k-1|k-1},P_{k-1|k-1}\right)$, and from this we obtain by Cholesky decomposition that $S_{k-1|k-1}$ = *chol* ($P_{k-1|k-1}$),

(1) Calculating and propagating cubature points

(13) $$X_{i,k-1|k-1}=S_{k-1|k-1}\xi_{i}+{\hat{x}}_{k-1|k-1}$$

(14) $$X_{i,\;k|k-1}^{*}=f(X_{i,\;k-1|k-1})$$

(15) $$\xi_{i}=\sqrt{\frac{m}{2}}\;{\left[1\right]}_{i}\;$$

where *m* is the number of volume points.

(2) Calculating the predicted value of states

(16) $${\hat{x}}_{\;k|k-1}=\frac{1}{m}\overset{m}{\underset{i=1}{\sum }}X_{i,\;k|k-1}^{*}$$

(3) Calculating the square root of the covariance matrix of the prediction error $W_{k}$

(17) $$S_{\;k|k-1}=\;\mathrm{Tria}\;\left(\left[X_{k|k-1}^{*}\;S_{Q,k-1}\;\right]\right)$$

where $S_{Q,k-1}$ represents the square root factor of $Q_{k-1}$, that is, $Q_{k-1}=S_{Q,k-1}S_{Q,k-1}^{T}$.

2 Measurement update

(1) Calculating and propagating volume points

(18) $$X_{i,\;k|k-1}=S_{\;k|k-1}\xi_{i}+{\hat{x}}_{k|k-1}$$

(19) $$Z_{i,\;k|k-1}=h(X_{i,\;k|k-1})$$

(2) Calculating the measured predicted value

(20) $${\hat{z}}_{\;k|k-1}=\frac{1}{m}\overset{2n}{\underset{i=1}{\sum }}Z_{i,\;k|k-1}$$

(3) Calculating the square root of the innovation covariance matrix

(21) $$S_{zz,k|k-1}=Tria\left(\left[\gamma_{k|k-1}S_{R,k}\right]\right)$$

where $S_{R,k}=chol\left(R_{k}\right)$, which means that $S_{R,k}$ is the square root factor of the measurement error covariance matrix $R_{k}$

(4) Calculating the square root of the cross-covariance matrix

(22) $$\chi_{k|k-1}=\frac{1}{\sqrt{m}}\left[X_{1,k|k-1}-{\hat{x}}_{\;k|k-1}\;\;X_{2,k|k-1}-{\hat{x}}_{\;k|k-1}\cdots X_{m,k|k-1}-{\hat{x}}_{\;k|k-1}\right]$$

(23) $$P_{xz,k|k-1}=\chi_{k|k-1}\gamma_{k|k-1}^{T}$$

(5) Estimating filter gain

(24) $$K_{k}=\left(P_{xz,k|k-1}/S_{zz,k|k-1}^{T}\right)/S_{\;zz,k|k-1}$$

(6) Status update

(25) $${\hat{x}}_{k|k}={\hat{x}}_{k|k-1}+K_{k}\left(z_{k}-{\hat{z}}_{\;k|k-1}\right)$$

(7) Calculating the square root factor of the state estimation error covariance matrix

(26) $$S_{\;k|k}=Tria\left(\left[\chi_{k|k-1}-K_{k}\gamma_{k|k-1}\;\;K_{k}S_{R,k}\right]\right)$$

### 3.3. Adaptive Square Root Cubature Kalman Filter Algorithm

In practical applications, due to continuous changes in environmental factors, the actual noise items will change accordingly. At this time, if the measurement noise covariance $R_{k}$ is always a fixed value, it may not reflect the actual situation of the received signal, resulting in key parameter values, such as position and speed, that cannot be accurately estimated. Therefore, it is necessary to realize real-time adjustment of the measurement noise covariance $R_{k}$ in the SRCKF algorithm and real-time estimation and correction of noise statistical characteristics in the filtering process to reduce state estimation errors. Based on the innovation sequence [14], this paper proposes an adaptive square root cubature Kalman filter algorithm to realize the adaptive adjustment of $R_{k}$ and compares the tracking results with the SRCKF VLL and ASRCKF SLL. The results prove that the algorithm proposed has a more accurate estimation performance and is more stable than the other two.

Since the observation is the output of the code phase discriminator and the carrier frequency discriminator, $R_{k}$ contains the code phase and carrier frequency errors. Normally, $R_{k}$ is set by calculating the variance of the discriminator output for a certain length of time. The longer this length of time, the more accurate the obtained variance, but if the length is set too long, the state value cannot be estimated in real time [20].

In Formula (32), $\left(z_{k}-{\hat{z}}_{\;k|k-1}\right)$ is defined as innovation. Therefore, the innovation is a linear combination of the observation vector and its predicted value. In an ideal state, the innovation is zero-mean Gaussian white noise, and the innovation at different moments should be orthogonal. However, in the target-tracking process, especially when the carrier is in the high dynamic state of motion, the observation noise will increase so that the innovation is no longer orthogonal. In order to solve the above problems, the adaptability of the algorithm must be improved [21].

The so-called adaptive filtering matches innovation with its theoretical covariance. Therefore, this paper applies the idea of covariance matching based on the innovation sequence to the square root cubature Kalman filter algorithm and realizes the adaptive adjustment of the measurement noise covariance matrix $R_{k}$ in the algorithm [16].

According to the literature [22,23], the measurement noise error covariance matrix $R_{k}$ can be estimated as

(27) $${\hat{R}}_{k}=E\left[(z_{k}-{\hat{z}}_{k|k-1})(z_{k}-{\hat{z}}_{k|k-1}){}^{T}\right]+\frac{1}{2n}\sum_{j=j_{0}}^{2n}z_{j,k|k}z_{j,k|k}^{T}-{\hat{z}}_{k|k}{\hat{z}}_{k|k}^{T}$$

where $z_{j,k|k}=h\left(X_{j,k|k}\right)$ is the volume point propagated through the function relationship of the measurement equation, $X_{j,k|k}=S_{\;k|k}\xi_{i}+{\hat{x}}_{k|k}$ is the measurement update cubature point, and ${\hat{z}}_{k|k}=\frac{1}{m}\sum_{i=1}^{2n}Z_{i,\;k|k}$ is the measurement prediction.

The covariance matrix of innovation is

(28) $$P_{ino}=E[(z_{k}-{\hat{z}}_{\;k|k-1}){\left(z_{k}-{\hat{z}}_{\;k|k-1}\right)}^{T}]$$

If a piece of sample data of innovation is used to approximate the covariance matrix of innovation [10], then there is

(29) $$P_{ino}\approx \frac{1}{N}\overset{N-1}{\underset{j=0}{\sum }}(z_{k-j}-{\hat{z}}_{\;k-j|k-j-1}){\left(z_{k-j}-{\hat{z}}_{\;k-j|k-j-1}\right)}^{T}$$

Therefore, we can get

(30) $$S_{R,k}=\sqrt{R_{k}}=\sqrt{\frac{1}{N}\overset{N-1}{\underset{j=0}{\sum }}(z_{k-j}-{\hat{z}}_{\;k-j|k-j-1}){\left(z_{k-j}-{\hat{z}}_{\;k-j|k-j-1}\right)}^{T}+}\;\frac{1}{2n}\overset{2n}{\underset{j=j_{0}}{\sum }}z_{j,k|k}z_{j,k|k}^{T}-{\hat{z}}_{k|k}{\hat{z}}_{k|k}^{T}$$

### 3.4. Realization of ASRCKF Algorithm in Vector Tracking Loop

#### 3.4.1. ASRCKF Vector Tracking Algorithm Implementation Specific Process

The receiver uses the RF front-end to down-convert the received signal into an intermediate frequency signal. After mixing, correlation, and other processes, the receiver outputs six coherent integration results of early, instant, and late, that is $I_{E},Q_{E},\;I_{P},Q_{P},I_{L},Q_{L}$, then uses the arctangent phase detection method for carrier to output carrier frequency difference and uses the noncoherent early minus late amplitude method for C/A code to output code phase difference. They are shown in Formula (31). The carrier frequency difference and code phase difference are used as measurements to input the navigation filter to monitor the loop tracking, and the filter uses the ASRCKF algorithm to calculate the position, velocity, acceleration, and other parameters.

(31) $$\emptyset_{carrier}=arctan\frac{Q_{P}}{I_{P\;}},\;\delta_{C/A}=\frac{1}{2}\frac{E-L}{E+L}$$

The receiver vector tracking loop (VTL) includes two parts: C/A code vector tracking and carrier signal vector tracking. Whether it is C/A code vector tracking or carrier signal vector tracking, it is necessary to first obtain the control amount of local pseudo code and carrier signal from the navigation filter. From the measurement in Equation (2), it is known that the matrix H is determined by the observation vector from the receiver to the satellite, which means that the position of the receiver and the satellite must be known to determine the matrix. Therefore, the receiver is first started in the traditional way; after obtaining the corresponding control amount through the code phase/carrier frequency estimator, it switches to the vector tracking mode. Through the tracking process of the vector loop, it can be known that if the signal of a certain channel suddenly disappears, because of the assistance of other channels in the C/A code vector tracking loop, the position error estimation of the C/A code loop through the user’s position feedback will always exist, and according to the estimated value, the phase of the local code is kept updated, so the loop will not lose lock in a short time. On the contrary, the scalar tracking loop will immediately enter the capture state to start the capture algorithm at this time, so the vector loop not only enhances anti-interference ability but also reduces a certain amount of calculation [16].

The Table 1 shows the initial settings of the relevant data of the vector loop.

#### 3.4.2. The Specific Implementation Steps of ASRCKF

1. Time update

(1) First, the SRCKF algorithm is used to obtain the state estimation value ${\hat{x}}_{\;k-1|k-1}$ and the square root of the state estimation error covariance matrix $S_{\;k-1|k-1}$ through the given model.

Among them $S_{\;k-1|k-1}=\sqrt{P_{\;k-1|k-1}}$. Let the process noise $W_{k}$ and the measurement noise $U_{k}$ be independent of each other, $W_{k}$ ~ $N\left(0,\;Q_{k}\right)$, $U_{k}$ ~ $N\left(0,\;Q_{k}\right)$.

The initial value of process noise covariance $Q_{0}=diag\left(1,1,1,1,1,1,1,1,1\right)$. The initial value of the measurement noise covariance $R_{0}=diag{\left(10,10\cdots \cdots 10\right)}_{2n\times 2n}$.

Use Equations (16) and (17) to calculate the cubature point and use the state equation to propagate it. The state transition matrix *F* is as follows:

(32) $$F=\left[\begin{array}{ccc} I_{3} & TI_{3} & \frac{1}{2}T^{2}I_{3}\\ 0_{3} & \;I_{3} & TI_{3}\\ 0_{3} & 0_{3} & I_{3} \end{array}\;\right]$$

*m* is the number of volume points. If *n* is used to represent the dimension of the state vector, then *m* = 2*n* is used in the SRCKF algorithm. Additionally, ${\left[1\right]}_{i}$ represents the *i*th column of matrix [1], and [1] represents the point set here:

(33) $$\left[1\right]={\left[\left(\begin{array}{c} 1\\ 0\\ \vdots \\ 0 \end{array}\right),\left(\begin{array}{c} 0\\ 1\\ \vdots \\ 0 \end{array}\right),\cdots ,\left(\begin{array}{c} 0\\ 0\\ \vdots \\ 1 \end{array}\right),\left(\begin{array}{c} -1\\ 0\\ \vdots \\ 0 \end{array}\right),\left(\begin{array}{c} 0\\ -1\\ \vdots \\ 0 \end{array}\right),\cdots ,\left(\begin{array}{c} 0\\ 0\\ \vdots \\ -1 \end{array}\right)\right]}_{9\times 9}$$

(2) Calculate the predicted value of the state ${\hat{x}}_{\;k|k-1}$ and $S_{\;k|k-1}$, which is the square root of the covariance matrix of the prediction error $W_{k}$.

Among them, $S_{Q,k-1}$ represents the square root factor of $Q_{k-1}$, and $Q_{k-1}$ represents the covariance matrix of the prediction error $W_{k-1}$.

2. Measurement update
  (1) The measured predicted value is obtained through Equations (24)–(26).
  (2) $S_{zz,k|k-1}$, the real-time square root of the innovation covariance matrix is obtained through Equation (30).
  (3) The real-time updated value of the state and square root factor of the state estimation error covariance matrix is obtained through Equations (22)–(26), and then enters the next cycle.

## 4. Simulation Test

In this experiment, the HWA-RNSS-7300 satellite navigation signal simulator of Beijing Huali Chuangtong Technology Co., Ltd. in Beijing, China.), was used to generate the Beidou satellite navigation signal required for the experiment. The IF (intermediate frequency) data collector of Xingyuan Beidou Navigation Technology Co., Ltd. in Beijing, China, was used to down-convert the output signal of the simulator into an IF signal. The output IF signal is $f_{IF}=4.092$ MHz, the sampling frequency is $f_{s}=16.368$ MHz, and the number of sampling bits is 2 bits. The receiver uses a vector software receiver, the pre-detection integration time is 1 ms, and the tracking threshold is 35 dB/Hz. Because it is difficult to generate real jerk signals and large-value acceleration signals, the highly dynamic model of the Jet Propulsion Laboratory (JPL) in the United States was used to simulate the motion state of the receiver.

**Figure 2:**
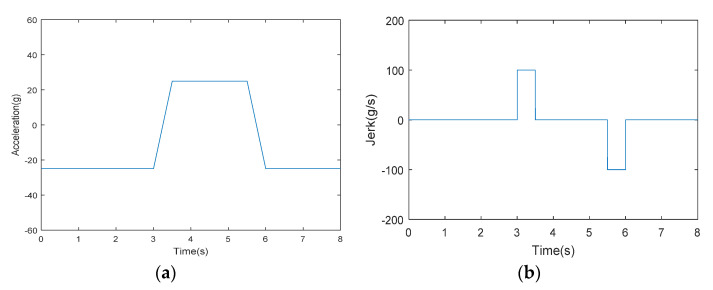
The trajectory of the receiver relative to the Beidou satellite. (**a**) Acceleration model for highly dynamic motion; (**b**) Jerk model for highly dynamic motion. Reprinted from ref. [24].

The highly dynamic movement of the receiver relative to the satellite will follow Figure 2a,b. Figure 2a is the acceleration signal model of the receiver, Figure 2b is the jerk signal model [24], and the simulation time is 8 s. At first, the jerk of the receiver was zero, and the receiver made a uniform acceleration movement at an acceleration of −25 g. However, the jerk suddenly increased to 100 g/s at 3 s and continued for 0.5 s. At this time, the acceleration of the receiver became 25 g and made a 2 s uniform acceleration motion with an acceleration of 25 g. At 5.5 s, the jerk suddenly changed to −100 g/s and lasted for 0.5 s; at this time, the acceleration became −25 g again, and uniform acceleration motion was made [25].

For the above-mentioned high dynamic model, this article uses the Discrete Wiener Process Acceleration (DWPA) [26,27] to model it. The process equation and the observation equation are respectively (1) and (2), and the process noise covariance matrix

(34) $$Q^{2}=\left[\begin{array}{ccc} \frac{1}{4}T^{4}I_{3} & \frac{1}{2}T^{3}I_{3} & \frac{1}{2}T^{2}I_{3}\\ \frac{1}{2}T^{3}I_{3} & \frac{1}{2}T^{2}I_{3} & TI_{3}\\ \frac{1}{2}T^{2}I_{3} & TI_{3} & I_{3} \end{array}\right]$$

Measurement noise covariance matrix

(35) $$R^{2}={\left[diag{\left(R_{code,1\;,\;}\;R_{carrier,1\;\;}\cdots \cdots \cdots R_{code,n\;,\;}\;R_{carrier,n}\right)}_{2n\times 2n}\right]}^{2}$$

The simulation first takes the root-mean-square error of the frequency as the evaluation index. Through 500 Monte Carlo experiments, the relationship curve between the RMS frequency error of the channel and the carrier-to-noise ratio CNR is obtained. The definition of RMSE is as follows:

(36) $$RMSE=\sqrt{\frac{1}{M}\overset{M}{\underset{i=1}{\sum }}{\left(\;X_{n}\left(k\right)-{\hat{X}}_{n}^{i}(k|k)\right)}^{2}}$$

The movement process of the carrier is as follows: the number of signal channels during 0 s–25 s is 12; during this period, the carrier moves in a straight line at a constant speed. After that, during 26 s−120 s, the number of channels with signals drops to three or fewer, which means that the carrier is in a weak signal environment at this time. During the period of 26−60 s, it is still moving in a straight line at a constant speed. Then, during 60−85 s, the carrier periodically performs a highly dynamic movement as shown in Figure 2; at this time, the receiver is in an extremely harsh high dynamic weak signal environment. After 120 s, the number of signal channels is restored to 12, and the uniform linear motion is restored.

**Figure 3:**
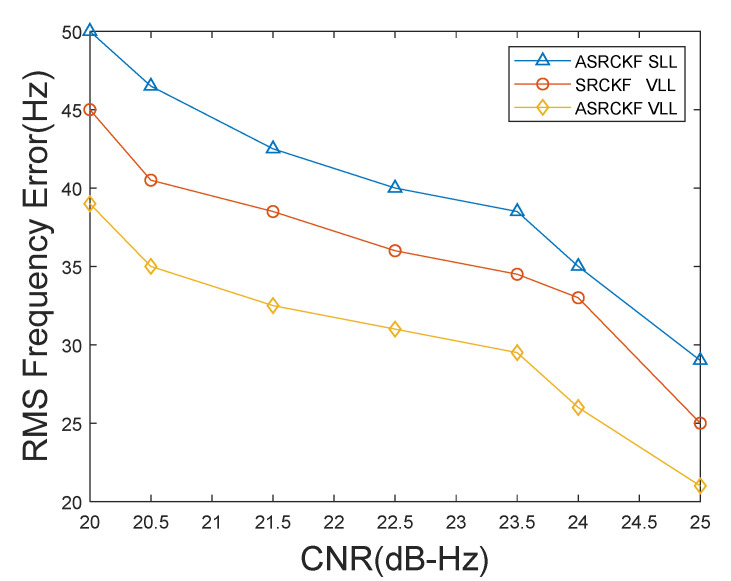
The relationship between RMS frequency estimation error and CNR.

Figure 3 shows the relationship between the RMS frequency error of channel 2 and the carrier-to-noise ratio (CNR). The frequency estimation value of channel 2 is obtained by multiplying the speed estimation value of the receiver by the scale factor μ [17].

(37) $$\mu =\frac{f_{B1}}{c}\approx 5.20366\;\;\mathrm{Hz}$$

Figure 3 shows the relationship curve between the RMS frequency error and CNR. It can be seen from Figure 3 that, compared to SRCKF VLL and ASRCKF SLL, the ASRCKF VLL has a significant improvement in frequency tracking accuracy. When C/N_0_ = 21.5, the tracking accuracy of the ASRCKF VLL is 6 dB higher than that of the SRCKF VLL and 10 dB higher than that of the ASRCKF SLL. When C/N_0_ = 22.5, the mean square error of the frequency estimation of the ASRCKF VLL is 31 Hz, which is 5 dB higher than that of the SRCKF VLL and 9 dB higher than that of the ASRCKF SLL, but as the carrier-to-noise ratio C/N_0_ increases, this improvement gradually decreases.

In addition, Figure 3 shows that the tracking performance of the SRCKF VLL is better than that of the ASRCKF SLL. This is because when the carrier-to-noise ratio is less than the tracking threshold, the signal strength is weak. In a weak signal environment, the weak signal channel of the vector tracking loop can be assisted by the strong signal channel, and the loop can achieve rapid recovery when the signal is blocked. Therefore, even if the SRCKF VLL cannot adaptively adjust the observed noise covariance matrix as the environment changes, it shows better tracking performance than the ASRCKF SLL.

**Figure 4:**
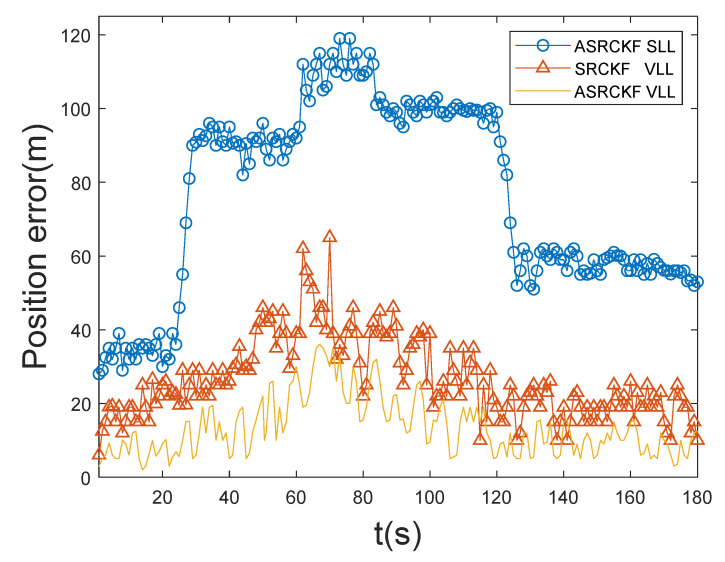
Position error.

**Figure 5:**
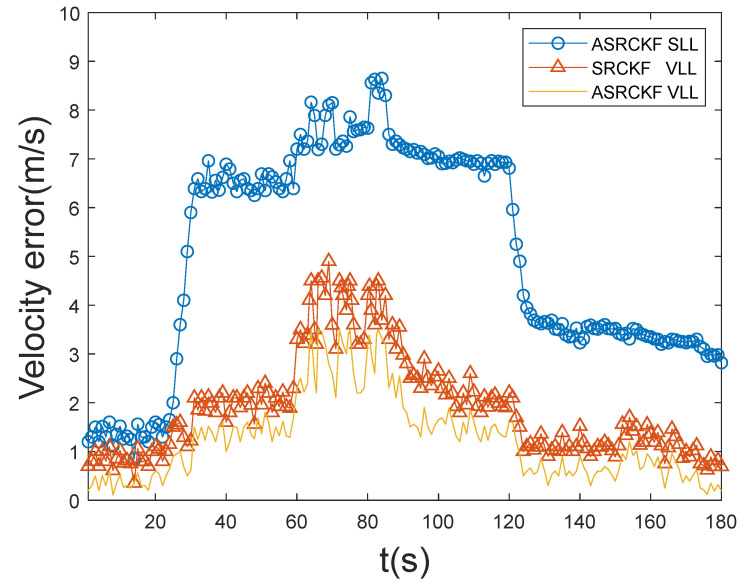
Velocity error.

From Figure 4 and Figure 5, it can be seen that the ASRCKF scalar tracking loop has good tracking performance in the initial 25 s, but during 25–120 s, the position error and speed error increase rapidly, and the tracking performance gap with the other two vector loops also increases rapidly; its tracking performance deteriorates further with the high dynamic movement of the receiver during 60–85 s. After 120 s, the number of channels with signals is restored to 12. At this time, the position error and speed error of the ASRCKF scalar loop are greatly reduced, but the tracking state before 25 s cannot be restored. The maximum position error gap with the ASRCKF VLL at this time is 82 m, and the maximum velocity error gap is 4.36 m/s.

The ASRCKF VLL and the SRCKF VLL can maintain normal operation during the entire 180 s. Under the highly dynamic and weak signal environment during 60–85 s, the three-dimensional position error of the SRCKF VLL does not exceed 65 m, the three-dimensional velocity error does not exceed 4.9 m/s, the three-dimensional position error of the ASRCKF VLL does not exceed 36 m, and the three-dimensional velocity error does not exceed 3.5 m/s. During the entire 180 s, among the three loops, the position error and velocity error of the ASRCKF vector tracking loop are the smallest, and the tracking performance is the best.

## 5. Algorithm Performance Analysis

In order to evaluate the performance of the filtering algorithm, the following two types of root-mean-square errors are introduced [18]:

(38) $$RMSE_{\mathrm{time}}=\sqrt{\frac{1}{M}\sum_{j=1}^{M}{\left({\hat{x}}_{k}^{j}-x_{k}^{j}\right)}^{2}},\;k=1,2,\cdots ,N$$

(39) $$RMSE_{total}=\sqrt{\frac{1}{N}\sum_{k=1}^{N}{\left({\hat{x}}_{k}^{j}-x_{k}^{j}\right)}^{2}},\;j=1,\;2,\cdots ,M$$

where ${\hat{x}}_{k}^{j}$ and $x_{k}^{j}$ are the estimated value and true value of the state variable in the jth Monte Carlo simulation at time k, N is the length of discrete time, M is the number of Monte Carlo simulations, the expression (38) describes the average convergence speed of the filtering algorithm in the simulation, and expression (39) describes the filtering performance of the filtering algorithm [18].

**Figure 6:**
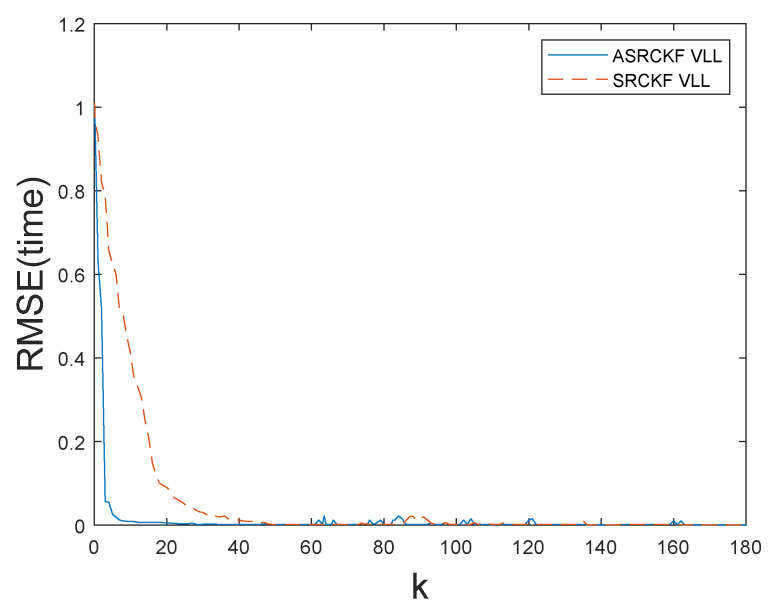
$RMSE_{\mathrm{time}}$ value when the filter uses the ASRCKF algorithm and the SRCKF algorithm to filter respectively and C/N_0_ = 30 dB-Hz.

The Figure 6 shows the corresponding $RMSE_{\mathrm{time}}$ value when the ASRCKF algorithm and the SRCKF algorithm are applied to the vector tracking loop when C/N_0_ = 30 dB-Hz.

Similarly, the $RMSE_{\mathrm{time}}$ value when C/N_0_ is 25, 23, 22, 21, and 19 dB-Hz, respectively, can be obtained. Table 2 describes the comparison of the filtering performance of the SRCKF and ASRCKF algorithms when the C/N_0_ is 25, 23, 21, 20, and 19 dB-Hz, respectively.

It can be seen from the Figure 6 and Table 2 that the numerical accuracy of the ASRCKF algorithm is higher than that of SRCKF, and the filtering performance of ASRCKF is better than that of SRCKF through the average value of $RMSE_{total}$. However, the time consumed is similar, that is, the computational complexity is similar. This result is consistent with the simulation results of Figure 4 and Figure 5.

## 6. Conclusions

In this paper, in order to improve the tracking performance of the vector receiver in the high dynamic and weak signal environment, the adaptive square root cubature Kalman filter algorithm is applied to the navigation filter in the vector tracking loop, which improves the stability and numerical accuracy of the filter, realizes the real-time measurement of the observed noise covariance matrix, and reduces the adverse effects caused by high dynamics and weak signal channels. The algorithm in this paper is compared with the vector loop based on SRCKF and the scalar loop based on ASRCKF. The test results show that when the number of signal channels is three or fewer, or even when the carrier performs high dynamic motion on that basis, compared with the SRCKF VLL and the ASRCKF SLL, the ASRCKF VLL shows lower position error and speed error and higher filtering accuracy. When the number of channels with signals is restored to four or more, the vector loop based on ASRCKF or SRCKF can achieve rapid retracking, while the scalar loop based on ASRCKF is only greatly improved compared to the highly dynamic weak signal. Compared with the other two vector loops, its tracking performance still has a gap. Additionally, through quantitative evaluation, the tracking performance of the ASRCKF algorithm in the vector tracking loop is about 18% higher than that of the SRCKF. Under the same conditions, the computational complexity of the two algorithms is similar. Therefore, in vector tracking loop, compared with the SRCKF algorithm, the ASRCKF algorithm can improve the tracking accuracy and tracking performance under the premise of the same computational complexity. In summary, the ASRCKF-based vector loop among the three loops has the most accurate estimation and tracking performance.

## Figures and Tables

**Table 1:** Initial settings of the relevant data of the vector loop.

Position Error—Variance (m^2^)	Velocity Error—Variance (m^2^/s^2^)	Code Phase Error—Mean Square Error	Carrier Frequency Error—Mean Square Error
10	2	12°	0.02 Chip

**Table 2:** The average time consumption and filtering performance of the two algorithms of ASRCKF and SRCKF when the C/N_0_ is 25, 23, 21, 20, and 19 dB-Hz, respectively.

Carrier-to-Noise Ratio (C/N0, dB/Hz)	Average Consumption Time of ASRCKF (s)	Average Consumption Time of SRCKF (s)	$\mathrm{Average}\;\mathrm{Value}\;\mathrm{of}\;RMSE_{total}$ of ASRCKF (s)	$\mathrm{Average}\;\mathrm{Value}\;\mathrm{of}\;RMSE_{total}$ of SRCKF (s)
25	0.0122	0.0123	0.0125	0.025
23	0.015	0.015	0.019	0.026
21	0.019	0.021	0.022	0.03
20	0.022	0.025	0.025	0.039
19	0.025	0.025	0.029	0.05

## Data Availability

Not applicable.

## Glossary

**List of symbols**	
V	velocity vector
$V^{\prime }$	acceleration vector
$V^{\prime \prime }$	Jerk vector
$X_{k}$	state, ‘*k*’ represents the time epoch of the data sample
$W_{k}$	state noise
$Z_{k}$	measurement
$U_{k}$	measurement noise
$z_{code}$	the output of code phase discriminator
$z_{carrier}$	the output of carrier frequency discriminator
$e_{j,k}$	line-of-sight direction unit vector between the receiver and the *j*th satellite at time epoch *k*
$R_{k}\;$	measurement error covariance matrix
$Q_{k-1}$	represents the covariance matrix of the prediction error $W_{k-1}$
$X_{k|k-1}^{*}$	represents the weighted center matrix,
$S_{zz}$	square root of the innovation covariance matrix
$S_{Q,k}$	square root factor of $\;Q_{k}$
$S_{R,k}$	square root factor of the measurement error covariance matrix $R_{k}$
$\gamma_{k|k-1}$	weighted center matrix
$K_{k}$	filter gain
${\hat{z}}_{\;k|k-1}$	measured predicted value
$S_{\;k|k}$	square root factor of the state estimation error covariance matrix
